# Parotid tuberculosis

**DOI:** 10.4103/0970-2113.71969

**Published:** 2010

**Authors:** Rajiv Garg, Sanjay Kumar Verma, Sumit Mehra, A N Srivastawa

**Affiliations:** *Department of Pulmonary Medicine, Chhatrapati Sahuji Maharaj Medical University, Uttar Pradesh, Lucknow, (erstwhile King George’s Medical University), India*; 1 Department of Pulmonary Medicine, G.S.V.M. Medical College, Kanpur, India; 2 Department of Pathology, Chhatrapati Sahuji Maharaj Medical University, Uttar Pradesh, Lucknow, (erstwhile King George’s Medical University), India

**Keywords:** Parotid gland, salivary gland, tuberculosis

## Abstract

Tuberculosis of the parotid gland is a rare condition. We describe a case of tuberculosis of right parotid gland in a 17-year-old male patient. Diagnosis was made by early suspicion and confirmed by demonstration of epitheloid granulomas on fine needle aspiration cytology (FNAC). Patient was successfully treated with daily regimen of four drugs (rifampicin, isoniazid, pyrazinamide, ethambutol) for first two months followed by two drugs (rifampicin and isoniazid) for last four months.

## INTRODUCTION

Parotid tuberculosis is a rare form of extra pulmonary tuberculosis. It is rare even in an endemic country like India.[1] Surprisingly parotid gland tuberculosis has very rarely been reported in the literature, only about hundred cases. It presents as a swelling in the parotid region. However if treated properly, the prognosis of tuberculosis of the parotid gland is good and surgery is not required in most of the cases. We report a case of primary tubercular parotiditis, in a patient who was diagnosed early and managed by a six-month regimen of antitubercular chemotherapy.

## CASE REPORT

**Figure 1:**
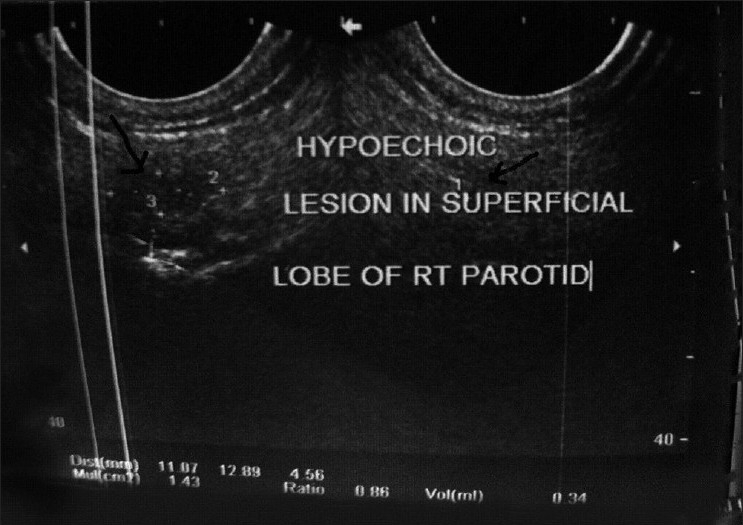
High-resolution sonography of the parotid region revealed a low level echo complex mass in superficial lobe of right parotid (size 32 × 17 mm) with no calcification or cystic degeneration, suggestive of inflammatory mass.

A 17-years-old male presented to us with a swelling of the right parotid region for last five months. The swelling was slow growing and was associated with toothache and difficulty in opening of the mouth with accompanied history of fever. There was no past history of tuberculosis in the family. No abnormality was found on physical examination. Local examination revealed a swelling in the right parotid region of size 3.0 × 1.5 cm, extending just below the pinna. The swelling was firm with ill-defined borders, mobile, with no attachment to the underlying bone or surrounding soft tissue. Local temperature was not raised and there was no scar mark or any sinus over the swelling. Facial nerve was intact, and the movements of cervical spine were also normal. No discharge or calculus in the region of the salivary ducts or any tonsillar enlargement was noted. There were multiple cervical lymph nodes in the neck. The rest of head and neck examination was normal. On investigation, his hemoglobin was 10.5 g%, total leukocyte count -7800/cmm^3^, differential leucocytes count: neutrophils 64%, lymphocytes 32% and eosinophil 4% and rest of the other investigations were normal. Chest radiograph was normal. High-resolution sonography of the parotid region revealed a low level echo complex mass in superficial lobe of right parotid (size 32 × 17 mm) with no calcification or cystic degeneration, suggestive of inflammatory mass [Figure 1]. Multiple lymph nodes were enlarged in right mid and upper cervical region and juglo digastric region was present. Mantoux test showed 20 mm induration. Sputum smear examination was negative for acid-fast bacilli (AFB) and enzyme linked immunosorbent assay (ELISA) for anti tubercular antibodies revealed; IgM-1195 Units (> 225 positive), IgG- 1.060 Units (>1 positive). Fine needle aspiration cytology (FNAC) of the swelling showed epitheloid granulomas and lymphohistiocytic clusters, suggestive of tubercular pathology. Ziehl-Neelsen (ZN) staining for AFB was, however, negative. Thus, the diagnosis of parotid tuberculosis was made. Patient was put on daily regimen of anti-tuberculosis treatment: four drugs (rifampicin, isoniazid, pyrazinamide, ethambutol) for first two months followed by two drugs (rifampicin and isoniazid) for last four months. There was marked response with this treatment, and swelling subsided after two months of therapy. He completed the maintenance phase successfully with disappearance of swelling at the end of treatment. He also gained 7 kg weight during treatment. He is presently asymptomatic on follow-up.

## DISCUSSION

Tuberculosis can involve any organ system in the body. While pulmonary tuberculosis is the most common presentation, extrapulmonary tuberculosis (EPTB) is also an important clinical problem.[2–4] Extra pulmonary tuberculosis (TB) represents approximately 25% of overall tubercular morbidity.[5] The term EPTB has been used to describe isolated occurrence of tuberculosis at body sites other than the lung. Among extra pulmonary tuberculosis (EPTB), the most common is lymph node tuberculosis while other forms are pleural tuberculosis, skeletal tuberculosis, CNS tuberculosis, abdominal tuberculosis, genito-urinary tuberculosis, miliary tuberculosis, and tubercular pericarditis.[6,7] Other less common forms of tuberculosis are cutaneous tuberculosis, otorhinolaryngeal tuberculosis, breast tuberculosis and disseminated tuberculosis.[8–11]

Parotid tuberculosis is a rare form of extra pulmonary tuberculosis. It usually presents as a unilateral swelling or abscess involving the parenchyma of the gland either through hematogenous spread or from infection of lymph nodes within or around it. It may also involve both the parotids.[12] C De Paoli reported the first case of parotid gland tuberculosis in 1893.[13] Since then, only about one hundred cases have been reported in the literature.[14] The most common route of infection of the parotid gland is by direct extension of the bacilli from the oral cavity via the gland ductal system.[15] An unusual form in which intra parotid and peri-parotid lymph nodes become infected either by lymphatic drainage from the oral cavity or hematogenously from a pulmonary focus is also known to occur. Parotid gland TB mostly presents as a localized and progressive chronic swelling. Clinical symptoms vary from an acute infectious process to an indolent chronic presentation.

Ultrasound represents the initial imaging modality of choice for the assessment of palpable abnormalities of the parotid gland and also of suspected parotid calculus disease.

The parotid glands are superficial structures and are readily amenable to high resolution ultrasound examination. Ultrasound is able to differentiate possible benign from malignant neoplasms; demonstrate whether a palpable lesion arises within the parotid gland, or is periparotid in location; and identify those entities that may not need surgical intervention.[16] Sonographic examination of the parotid swelling contributes substantially in the diagnosis of parotid TB infection. Sonographically parotid tuberculosis can be of two types namely parenchymal and periparotid type. The parenchymal type appears as a diffusely enlarged, comparatively hypoechoic gland, with or without focal intraparotid nearly anechoic zones, which might have a cavity or cavities within it as seen in the present case. The periparotid type appears as hypoechoic nodules located in the peripheral zone of the hyperechoic parotid gland, consistent with enlarged periglandular lymph nodes.[7] USG-guided fine needle aspiration cytology correlates well with postoperative histological findings and has an overall accuracy of 86-89%.[17,18]

The differential diagnosis includes generally benign malignant neoplastic diseases of the parotid and sarcoidosis.

Four drug regimen (rifampicin, isoniazid, ethambutol and pyrazinamide) in the intensive phase followed by two drugs (rifampicin and isoniazid) in continuation phase is a recommended treatment regimen.[19] However, a regimen consisting of only three drugs (rifampicin, isoniazid and pyrazinamide) followed by two drugs (rifampicin and isoniazid) may also be sufficient as it is a pauci-bacillary extra pulmonary form of tuberculosis. The early diagnosis and suspicion is required to avert the need for surgery which may be a hazardous procedure in a medically treatable condition.
